# Ethnic and socioeconomic inequalities in the mental health of children and young people with pre-existing mental health and neurodevelopmental conditions during the COVID-19 pandemic: a systematic review of longitudinal studies

**DOI:** 10.1007/s00787-026-02971-2

**Published:** 2026-01-31

**Authors:** Ann HY Lee, Johnny Downs, Valeria Parlatini, Shuo Zhang, Emily Simonoff, Brian CF Ching

**Affiliations:** 1 Department of Child and Adolescent Psychiatry, Institute of Psychiatry, Psychology and Neuroscience, King’s College London, London, United Kingdom; 2 National Institute for Health Research (NIHR) Biomedical Research Centre, South London and Maudsley NHS Foundation Trust, London, United Kingdom; 3 CAMHS Digital Lab, Department of Child and Adolescent Psychiatry, Institute of Psychiatry, Psychology and Neuroscience, King’s College London, London, United Kingdom; 4 Centre for Innovation in Mental Health, School of Psychology, University of Southampton, Southampton, United Kingdom

**Keywords:** COVID-19, Mental health, Neurodevelopmental, Children and young people, Ethnic and socioeconomic inequalities.

## Abstract

The COVID-19 pandemic may have amplified existing inequalities and disproportionately impacted the mental health of children and young people (CYP) with pre-existing mental health and neurodevelopmental conditions. Evidence suggests the mental health impact on this clinical group was heterogeneous, but the role of ethnicity and socioeconomic position on longitudinal mental health outcomes is unclear. This systematic review investigates the longitudinal association between ethnic and socioeconomic inequalities and the mental health outcomes of CYP with pre-existing conditions during the pandemic. OVID Medline, EMBASE, APA PsycInfo, and Global Health databases were searched between January 2020 and November 2025 (PROSPERO CRD42024611865). Eligible papers included longitudinal studies that assessed mental health outcomes at multiple timepoints before and/or during the pandemic in CYP with pre-existing conditions and examined the effect of ethnicity and socioeconomic position on outcomes. Included studies were narratively synthesised. Ten studies (*N* = 3,887) were included. We found evidence that CYP from lower income brackets and who experienced financial hardship reported greater levels of internalising, neurodevelopmental, post-traumatic stress, and obsessive-compulsive symptoms. Weak associations were found between ethnicity and internalising symptoms. However, the findings were inconsistent across mental health outcomes, timepoints, and ethnic and socioeconomic position groups. There is some evidence for the association between lower socioeconomic position and increased mental health outcomes in CYP with pre-existing conditions during the pandemic. Further robust longitudinal research is warranted to examine the long-term consequences of the pandemic to better understand how ethnic and socioeconomic inequalities contribute to mental health outcomes.

**Supplementary information:**

The online version contains supplementary material available at 10.1007/s00787-026-02971-2.

## Introduction

The COVID-19 pandemic significantly disrupted the lives of children and young people (CYP), including disruptions to education, social relationships, and family dynamics [1], often coinciding with critical developmental milestones such as educational transitions, the development of peer relationships, and social skills [2]. Rates of depression and anxiety in CYP in the general population may have doubled since the pandemic began [3], and their mental health has worsened during the pandemic compared to before [4].

CYP with pre-existing mental health or neurodevelopmental conditions may have been particularly vulnerable to adverse pandemic effects. The pandemic may have compounded pre-existing difficulties, such as special education needs, limited access to mental health services, and heightened stress [5]. A systematic review and meta-analysis of longitudinal studies of clinical groups of CYP found that the longitudinal impact of the pandemic on CYP with pre-existing conditions varied widely, shaped by individual and contextual vulnerabilities that were often overlooked in pooled analyses [6]. Individual studies reported some increases in internalising, externalising, and other symptoms in CYP with pre-existing conditions, though explanatory factors that may drive this were unclear [7–9]. Internalising symptoms typically refer to inwardly directed emotional difficulties such as anxiety, low mood, and social withdrawal, whereas externalising symptoms involve outwardly directed behaviours including aggression, hyperactivity, and conduct problems [10]. These domains are widely used to characterise CYP mental health, sensitive to environmental stressors, and are strong predictors of later mental health and functional outcomes [11].

CYP with neurodevelopmental conditions, including autism spectrum disorder (ASD) and attention deficit hyperactivity disorder (ADHD), often require more specialised support, which was less accessible during the pandemic [12]. These CYP typically depend on structured educational environments, routine-based support, and tailored interventions such as behavioural therapy, speech and language therapy, and occupational therapy [13]. Many also rely on consistent peer interactions and community programmes to support social communication and emotional regulation [14]. Pandemic-related school closures, service disruptions, and social distancing measures significantly reduced access to these essential supports, potentially exacerbating difficulties for CYP with neurodevelopmental conditions [15].

Ethnicity and socioeconomic position are well-established risk factors for poor mental health outcomes in CYP [16] and may also be associated with worse mental health outcomes in CYP during the pandemic. Black and Mixed CYP in the United Kingdom had higher rates of mental health diagnoses and psychotropic prescriptions during the pandemic, while Pakistani, Bangladeshi, and Black Caribbean children showed higher rates of internalising or externalising difficulties compared to White counterparts [17, 18]. Similarly, studies in the United Kingdom found that significant disparities in financial stress and employment opportunities for parents, which disproportionately affected lower-income families, may have widened during the pandemic [19, 20]. A previous cross-sectional analysis of data from the CYPHER survey, a clinical cohort study conducted in the United Kingdom, found parental concerns with housing and finances were associated with increased emotional difficulties in CYP with mental health and neurodevelopmental conditions, as reported by both young people and their parents [21]. However, they did not find consistent associations between ethnic groups and emotional or behavioural symptoms, further highlighting the complexity of these associations.

Despite growing research on the pandemic’s impact on CYP’s mental health, significant gaps remain in understanding what role ethnic and socioeconomic inequalities play in the mental health of CYP with pre-existing conditions longitudinally. In a systematic review of longitudinal studies on the mental health impact of the pandemic on CYP with pre-existing conditions, Ching and colleagues [6] found no consistent evidence that ethnic and socioeconomic differences explained variation in mental health outcomes in this clinical group. However, the included data in the review at the time spanned 2020–2021 and only a few studies examined these factors, which limited the analysis of these associations. Further investigation with more recent research is warranted to consider the long-term effects of ethnic and socioeconomic disparities in this clinical group. We hypothesised that CYP from minoritised ethnic groups and lower socioeconomic backgrounds would experience greater worsening of mental health outcomes from pre- and during the pandemic. Thus, this systematic review investigates the longitudinal association between ethnic and socioeconomic inequalities and mental health outcomes in CYP with pre-existing conditions during the pandemic, offering a more comprehensive and updated synthesis of the evidence to inform future research and policy.

## Methods

This systematic review was preregistered on PROSPERO (CRD42024611865) and followed the Preferred Reporting Items for Systematic Reviews and Meta-Analyses (PRISMA) guidelines [22].

### Eligibility criteria

We included longitudinal studies that presented data on CYP aged 3 to 18 years. Studies were eligible if CYP were clinically diagnosed with mental health or neurodevelopmental conditions by a clinician using the Diagnostic and Statistical Manual of Mental Disorders (DSM) [23] or International Classification of Diseases (World Health Organization) [24]; met clinical thresholds on validated measures such as the Strengths and Difficulties Questionnaire (SDQ) [25] and Paediatric Symptom Checklist (PSC) [26]; or were attending mental health services. We included any studies that measured the effects of ethnicity and/or socioeconomic position on mental health outcomes between pre- and during pandemic and/or between during pandemic phases. A comparator was not specified at the review level as study inclusion focused on longitudinal mental health outcomes in CYP with pre-existing conditions, irrespective of whether individual studies used internal comparison groups in their analyses. Eligible studies needed to be peer-reviewed and published in English. Grey literature, including preprints and conference abstracts, were excluded to ensure the reliability and quality of the included studies. See Appendix S1 for the Population, Exposure, Comparator, and Outcome (PECO) elements table.

### Search strategy

OVID Medline, EMBASE, APA PsycInfo, and Global Health were searched between 1 January 2020 and 25 November 2025 (see Appendix S2 for search terms). Relevant studies were additionally sourced through citation searching and manual searching via Google Scholar.

### Selection process

All eligible papers were double-screened independently using EndNote21 at the title/abstract and full-text screening stages (AL and BCFC). The inter-rater reliability demonstrated strong agreement for both title/abstract (*kappa* = 0.829) and full-text stages (*kappa* = 0.806). Discrepancies were resolved through discussions between the researchers.

### Data extraction

Data were double extracted independently (AL and BCFC), including study and sample characteristics (i.e., sample size, location, age, gender, ethnicity, socioeconomic position; indicators included socioeconomic status, family income, and financial hardship), measurement tools, assessment timepoints during the pandemic, COVID-19 restriction levels, mental health outcomes, and key findings.

### COVID-19 restriction levels

The Oxford COVID-19 Government Response Tracker (COVID-19 Stringency Index) [27] was used to evaluate restriction levels based on national pandemic data. This tracker provides standardised scores reflecting the severity and scope of government-imposed measures to control the pandemic at a national level.

### Risk of bias

We utilised an adapted scale based on multiple quality appraisal tools informed by published reviews of longitudinal studies [6]. AL and BCFC independently conducted the risk of bias assessments and resolved discrepancies through discussion (see Appendix S3 for detailed information on the risk of bias indicators).

### Synthesis methods

We conducted a narrative synthesis of all the studies and relevant outcomes to assess the impact of ethnicity and socioeconomic position on CYP’s mental health outcomes during the pandemic. Eligible mental health outcomes were those assessed using validated, standardised, or widely used clinical or behavioural measures of internalising, externalising, neurodevelopmental, or related symptom domains; studies relying solely on unvalidated or bespoke measures were excluded. The direction and magnitude of effects were highlighted where possible. Study findings were organised by ethnicity and socioeconomic position. Mental health outcomes were grouped into broader symptom categories: internalising symptoms, including depressive and anxiety symptoms; neurodevelopmental symptoms, including ASD and ADHD symptoms; and other symptoms, including post-traumatic stress and obsessive-compulsive symptoms, which are classified as distinct to internalising disorders in diagnostic frameworks [16]. For each study, we extracted the effect estimates reported, noting adjusted estimates where available; unadjusted estimates were extracted when no adjusted values were provided.

Studies were also grouped by assessment timepoints, comparing mental health outcomes between pre- and during pandemic, and between various pandemic phases. To allow for meaningful comparisons, we also categorised pandemic phases into acute (March 2020-June 2020), remission (July 2020-December 2020) and resurgence phases (January 2021-June 2021). No included studies provided longitudinal data beyond June 2021; therefore, the review synthesised findings within these three phases.

## Results

### Summary of included studies

**Fig. 1:**
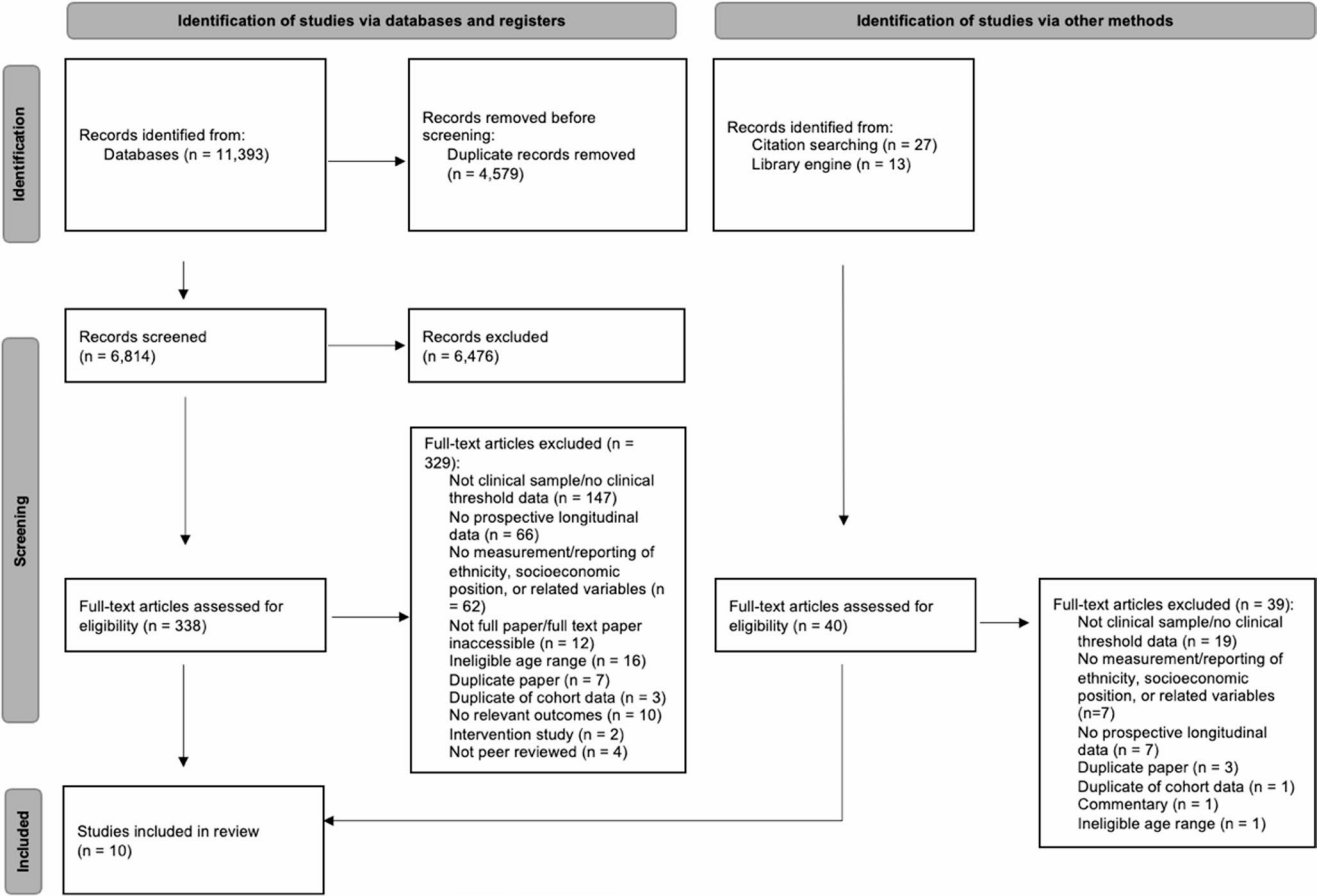
PRISMA flowchart

We identified 11,393 records, and 10 studies (*N* = 3,887) met the inclusion criteria (see Fig. 1 for PRISMA flowchart).

Studies were conducted in the United States (three), United Kingdom (one), Italy (one), Spain (one), Turkey (one), Canada (one), China (one), and India (one). Sample sizes varied (*N* = 34 to 1,393) across diagnostic groups, including depression and anxiety, ASD, ADHD, mixed neurodevelopmental conditions, and other neuropsychiatric conditions. Follow-up durations ranged from 2 to 26 months.

Regarding assessment timepoints, two studies assessed pre- and during pandemic mental health outcomes, while eight studies assessed mental health outcomes at multiple pandemic timepoints. During the pandemic, two studies assessed mental health outcomes in the acute phase (March-June 2020), six studies in the remission phase (July-December 2020), and two studies in the resurgence phase (January-June 2021). The COVID-19 Stringency Index for most countries during the study period ranged from 60 to 80, indicating moderate to high restriction levels [27]. Across the included studies, the statistical effect sizes differed in form, including adjusted and unadjusted regression coefficients, t-tests, adjusted prevalence ratios, and relative risk ratios. Studies also varied in the covariates included in adjusted models. See Table 1 for more details of the characteristics of included studies and Table 2 for a summary of key findings.

**Table 1:** Characteristics table of included studies

Author (year)	Country	Baseline *n* and sample		Baseline mean age (SD), range	Sex/Gender	Ethnicity	Socioeconomic positions (SES)	Pre-COVID timepoints	During Covid timepoints	Duration of follow-up (attrition rate)	The COVID-19 Stringency Index	Relevant outcomes and measures	Informant
Conti et al. (2020)	Italy	141 patients from a neuropsychiatric hospital	1.5–18		117 boys, 24 girls	Ethnicity: No data	Financial hardship: no extractable data	September 2019-February 2020	April-May 2020	~ 2–8 months	80	Depressive, anxiety, conduct problems (CBCL)	Parent
Dvorsky et al. (2022)	United States	118 adolescents with ADHD from public schools	15–17		132 boys, 106 girls. (gender distribution reported for the entire sample)	Ethnicity: 82% White, 7% multiracial, 6% Black, 4% Asian, 4% Latinx	Family Income: 19% below U.S. median income.	N/A	May-June 2020; July-Aug 2020; Oct-Nov 2020	~ 6 months	65; 65; 65	Depressive and anxiety symptoms (CRISIS)	YP
Estrada-Prat et al. (2024)	Spain	418 children with elevated emotional and behavioural symptoms from EmCoVID19 study	7.76 (2.92), 4–17		244 boys, 174 girls	Ethnicity: No data	SES: 1.46% lower, 4.62% lower-middle, 11.19% middle, 36.98% middle-upper, 45.74% upper class.	N/A	June 2020-Feb 2021 (Monthly assessments)	~ 9 months (10% retention rate by the final timepoint)	70	Emotional and behavioural symptoms (PSC)	Parent
Kaya et al. (2024)	Turkey	42 autistic children from a teaching hospital	4.69 (1.09), 3–6		34 boys, 8 girls.	Ethnicity: No data	Family Income: 59.5 <4000 TL, 40.5% >4000 TL	Jan-March 2020	June-Sept 2020	~ 3–9 months (N/A)	70	ASD severity (CARS)	Clinician
Korczak et al. (2024)	Canada	932 clinically referred children/adolescents with neurodevelopmental or mental health issues	Parent Report: 11.79 (2.90), 8–18; Child Report: 12.91 (2.48), 10–18; Parent Report: 4.37 (1.37), 2–7		Parent Report (8–18 years): 524 boys, 407 girls, 1 other	Ethnicity: 66% European, 16.7% Non-European (single ancestry), 15.1% multiple ancestries, 2.1% no response	Family Income: 23.3% <$80,000, 61.8% >$80,000, 14.9% no response	N/A	April-June 2020; July-Aug 2020; Sept- Nov 2020; Dec 2020-May 2021; June-Oct 2021; Nov 2021-March 2022; April to June 2022	~ 22–26 months (54% retention rate by the fifth timepoint)	75;70; 70; 75; 70; 70; 30	Depressive symptoms (RCADS), anxiety symptoms (SCARED), emotional and behavioural problems (SDQ)	Parent and YP
Oblath et al. (2024)	United States	172 children from a safety-net hospital (general population study)	8.5 (1.8), 6–11		97 boys, 75 girls	Ethnicity: 54.7% Non-Latino Black, 27.9% Latino, 5.2% White, 1.7% Other, 10.5% Unknown	Difficulty Paying Bills: 15.8% of families	N/A	Aug 2020-Jan 2021; Dec 2020-March 2021; March–July 2021 (Approximate waves due to paper not being explicit)	~ 6 ~ 11 months (75% retention rate across all timepoints)	67; 70; 65	Emotional and behavioural symptoms (PSC-17) and social risks (THRIVE)	Parent
Pampati et al. (2023)	United States	1,561 from the COVID Experiences Survey; 322 with pre-existing conditions (general population study)	5–12		779 boys, 725 girls, 57 not stated (gender distribution reported for the entire sample)	Ethnicity: 55.2% White non-Hispanic, 11.3% Black non-Hispanic, 23.1% Hispanic, 10.4% Other non-Hispanic	Family Income: 22.3% <$30,000, 77.7% ≥$30,000	N/A	October-November 2020; March-May 2021	~ 4–7 months (82% retention rate by second timepoint)	65; 65	Child depressive, anxiety and psychological symptoms (PROMIS)	Parent
Rong et al. (2024)	China	1,393 junior high school students (general	13.83 (0.82), 12–16		743 boys, 650 girls	Ethnicity: 98.9% Han Chinese	COVID impact on finance : 37.0% no impact, 33.8% mild, 22.6% moderate, 4.3% severe, 2.3% extremely severe	N/A	April-July 2020 (Specific dates: April 22–28, May 6–8, May 27–30, June 16–20, June 29-July 2)	~ 3 months (85.1% retention rate by the final timepoint)	80	Depressive symptoms (PHQ-9) and anxiety symptoms (GAD-7)	YP
Saurav et al. (2023)	India	34 children with ADHD from outpatient services	Jun-15		20 boys, 14 girls	Ethnicity: No data	SES: 5.88% lower, 38.23% upper-lower, 35.29% lower-middle, 20.59% upper-middle, 0% upper	N/A	July-Sept 2022	~ 2 months	40	ADHD symptoms (CPRS)	Parent
Toseeb et al. (2022)	United Kingdom	517 autistic young people	10.69 (3.34), 5–18		352 boys, 152 girls. 13 not stated	Ethnicity: 90% White British, 10% ethnic minorities	Family Income: 48% below median	N/A	March-April 2020; April 2020-May 2020; May 2020-June 2020; Sept-Oct 2020	~ 6 months (63% retention rate by the final timepoint)	60; 60; 65; 60	Depressive and anxiety symptoms (RCADS)	Parent

Note. SES = Socioeconomic Status; SD = Standard Deviation; CBCL = Child Behaviour Checklist; CRISIS = Ten-item Mental Health Subscale of the Coronavirus Health Impact Survey; PSC = Paediatric Symptom Checklist; CARS = Childhood Autism Rating Scale; RCADS = Revised Child Anxiety and Depression Scale; SCARED = Screen for Child Anxiety Related Disorders; SDQ = Strengths and Difficulties Questionnaire; THRIVE = Tool for Health and Resilience in Vulnerable Environment; PROMIS = The Patient Reported Outcomes Measurement Information System; PHQ-9 = Nine-item Patient Health Questionnaire; GAD-7 = Generalised Anxiety Disorder Scale; CPRS = Conners’ Parent Rating Scale

**Table 2:** Key findings of included studies

Author (year)	Key findings
Conti et al. (2020)	(1) Ethnicity: not reported. (2) Financial hardship experienced by families during April-May 2020 (acute) was significantly associated with an increase in post-traumatic stress (*β* = −2.540, *p* <.05) and obsessive-compulsive symptoms (*β* = −3.248, *p* <.05) in the 6–18 years subpopulation. Analyses adjusted for age, diagnosis, and baseline mental health symptom levels. Note. Negative beta coefficients in this study represent increases in symptom severity because the authors modelled change scores as pre-pandemic minus during-pandemic symptoms. Thus, more negative values reflect worsening symptoms
Dvorsky et al. (2022)	(1) Ethnicity was significantly associated with mental health symptom severity (*p* =.010), such that for those who identified as Black were associated with greater severity of symptoms from May-November 2020 (acute and remission) (2) Socioeconomic position: not reported. Analyses adjusted for age, sex, ethnicity, parent education, family income, and for baseline mental health symptoms.
Estrada-Prat et al. (2024)	(1) Ethnicity: not reported. (2) There was no significant impact of socioeconomic status on children’s psychosocial problems during June 2020-February 2021 (acute, remission and resurgence), (*β* = − 0.30 to 0.87, *p* >.05). Study did not specify whether analyses were adjusted
Kaya et al. (2024)	(1) Ethnicity: not reported. (2) The severity of autism symptoms increased significantly more in June-September 2020 (remission) compared to March 2020 (acute) in children from families with a monthly income of less than 4,000 TL than in those with higher family incomes (*t*(40) = −3.09, *p* <.001). Analyses were unadjusted
Korczak et al. (2024)	(1) Ethnicity did not significantly predict individual differences in mental health outcomes for depressive symptoms (*β* = 0.95, *SE* = 1.80, *p* =.60) or anxiety symptoms (*β* = − 0.33, *SE* = 0.54, *p* =.54) during April 2020-June 2022 (acute to resurgence). (2) Family income did not significantly predict individual differences in mental health outcomes for depressive symptoms (*β* = − 0.88, SE = 1.54, *p* =.57) and anxiety symptoms (*β* = − 0.12, SE = 0.47, *p* =.81) during April 2020-June 2022 (acute to resurgence). Analyses adjusted for baseline mental health symptoms
Oblath et al. (2024)	(1) Ethnicity: not reported. (2) The difficulty of paying bills significantly increased during all waves compared to pre-pandemic levels (*p* <.001), assessed in August 2020-July 2021 (remission to resurgence). Overall, social risks were significantly associated with emotional and behavioural problems across all waves (*p* <.001). Analyses adjusted for child age, sex, and caregiver ethnicity, but not baseline mental health symptoms
Pampati et al. (2023)	(1) Ethnic minority children had a higher likelihood of experiencing clinically significant depressive and anxiety symptoms compared to White, non-Hispanic children in November 2020 (remission) and May 2021 (resurgence), but these differences were not statistically significant (*β* range: − 0.9 to 0.3, *p* >.05, 95% CI [−3.2, 2.0]). (2) Children from households with an annual income below $30,000 were significantly more likely to exhibit clinically elevated depressive, anxiety, and stress symptoms compared to those from higher-income households in November 2020 (remission) and May 2021 (resurgence) (APR = 1.5, 95% CI [1.0, 2.2], *p* <.05). Analyses were adjusted for child sex, grade level, and health insurance status, but not for baseline mental health symptoms
Rong et al. (2024)	(1) Ethnicity: not reported. (2) The perceived negative impact of COVID-19 on family income was a significant predictor of higher severity for depressive and anxiety symptoms in April-June 2020 (acute). For depressive symptoms, the perceived negative impact of COVID-19 on family income was significantly associated with chronic dysfunction (RRR = 1.39, 95% CI: 1.09, 1.77). For anxiety, the perceived negative impact on family income was significantly associated with chronic dysfunction (RRR = 1.57, 95% CI: 1.16, 2.14). Analyses adjusted for sex and grade level, but not for baseline mental health symptoms
Saurav et al. (2023)	(1) Ethnicity: not reported. (2) No significant associations were found between socioeconomic status and ADHD symptoms pre- and post-lockdown in April 2020 (acute; *p >*.05). Analyses were unadjusted
Toseeb et al. (2022)	(1) Ethnic minority children exhibited slightly lower depressive (*β* = − 0.89, 95% CI [−2.39, 0.61], *p* >.05) and anxiety symptoms *β* = −3.34, 95% CI [−7.19, 0.51], *p* >.05) compared to White British children, though these differences were not statistically significant during March-October 2020 (acute to remission). (2) Children from below-median-income households had significantly higher anxiety symptoms (*β* = 2.52, 95% CI [0.22, 4.82], *p* <.05), but the higher depressive symptoms observed did not reach statistical significance *β* = 0.32, 95% CI [−0.58, 1.22], *p* >.05) during March-October 2020 (acute to remission). Analyses adjusted for child age, sex, school type, and household income, but not for baseline mental health symptoms

Note. ADHD = Attention Deficit Hyperactivity Disorder; ASD = Autism Spectrum Disorder; SES = Socioeconomic Status; CI = Confidence Interval; RRR = Relative Risk Ratio; APR = Adjusted Prevalence Ratio; SD = Standard Deviation; SE = Standard Error PHQ-9 = Patient Health Questionnaire-9; GAD-7 = Generalised Anxiety Disorder-7; RCADS = Revised Child Anxiety and Depression Scale; CARS = Childhood Autism Rating Scale; CPRS = Conners’ Parent Rating Scale; PROMIS = Patient-Reported Outcomes Measurement Information System

### Risk of bias

Eight studies exhibited a moderate risk of bias due to concerns such as sample size, outcome assessment, and confounding variables. Two studies showed a low risk of bias across most of the assessed domains. None of the studies were rated with a high risk of bias. See Appendix S4 for detailed risk of bias assessment ratings.

### Ethnicity

The relationship between ethnicity and CYP’s mental health outcomes was examined in four studies, with participant demographics varying by study location and cultural context. All four studies were conducted in Western countries (e.g., the United States and United Kingdom). Across these studies, participants were predominantly White (55%−90%), comprising non-minoritised ethnic groups in their respective settings, while smaller proportions were from ethnically minoritised groups, including Black (6%−11%), Hispanic/Latinx (4%−23%), Asian (4%), mixed (7%−16%), and other ethnicities (1%−10%).

#### Internalising symptoms

Four studies assessed the relationship between ethnicity and internalising symptoms during the pandemic. Studies included children with depression and anxiety (one study), autistic children (one study), children with ADHD (one study), and children and adolescents from a general population study with elevated emotional and behavioural symptoms (one study). Two studies used parent-reported data, one study used CYP-reported data, and one study used both parent and CYP-reported data. Across these four studies, two adjusted for baseline mental health symptoms [28, 31], while the remaining two analysed only during-pandemic symptom levels without baseline adjustment [29, 30].

For depressive and anxiety symptoms, most studies reported no significant associations with ethnicity across the acute, remission, and resurgence phases of the pandemic. In Canada, ethnicity was not significantly associated with depressive (*β* = 0.95, SE = 1.80, *p* =.60) or anxiety symptoms (*β* = − 0.33, SE = 0.54, *p* =.54) in children with depression and anxiety after adjusting for baseline symptoms, in a sample predominantly comprising White European participants [28]. Pampati and colleagues [29] similarly found no significant differences in depressive or anxiety symptoms among Black, Hispanic, and other non-Hispanic children with elevated emotional and behavioural problems compared to White children in the United States (*β* range: − 0.9 to 0.3, *p* >.05, 95% CI [−3.2, 2.0]). While analyses accounted for demographic variables such as age, sex, and the presence of behavioural or physical conditions, baseline mental health symptoms were not adjusted for. In the United Kingdom, Toseeb and colleagues [30] found no significant differences in depressive (*β* = − 0.89, 95% CI [−2.39, 0.61], *p* >.05) or anxiety symptoms (*β* = −3.34, 95% CI [−7.19, 0.51], *p* >.05) between autistic children from minoritised ethnic backgrounds and White British children. Analyses only adjusted for sociodemographic variables, including household income, sex, and school type. In contrast, Dvorsky and colleagues [31] reported significantly greater symptom severity for both depressive and anxiety symptoms among Black adolescents with ADHD compared to White and Asian counterparts after adjusting for baseline symptom severity (*p* =.01) across the acute and remission phases of the pandemic in the United States.

### Socioeconomic position

The relationship between socioeconomic position and CYP’s mental health outcomes was examined in nine studies using indicators including family income (five studies), socioeconomic status (SES; two studies), and financial hardship (two studies). Most data on socioeconomic positions were parent-reported, with one study using CYP-reported data. Two studies used standardised scales to assess socioeconomic positions; one study used Tool for Health and Resilience in Vulnerable Environments (THRIVE), which assessed financial hardship, and another study used Coronavirus Health and Impact Survey (CRISIS), which measured socioeconomic position and other pandemic-related variables. Other studies collected socioeconomic data via general demographic questionnaires. Most CYP were from middle-class backgrounds (35.3%−61.8%), with fewer from lower-class (1.5%−38.2%) and upper-class (0%−45.7%) households. CYP from families earning below-median income comprised 19%−59.5% of participants, and 15.8% of families reported experiencing financial hardship across the studies.

#### Internalising symptoms

Six studies assessed the relationship between socioeconomic position and internalising symptoms. Studies included children with depression and anxiety (one), autistic children (one), and children from general population studies with elevated emotional and behavioural symptoms (four). Three studies used parent-reported data, one used CYP’s data, and one used both parent and young people-reported data. Among the six studies assessing socioeconomic position and internalising symptoms, one adjusted for baseline mental health levels [28], while four adjusted for sociodemographic factors but not for baseline symptoms [29, 30, 32, 34], and one did not report whether baseline adjustment was performed [33].

Findings on the relationship between socioeconomic position and depressive and anxiety symptoms varied. Most studies reported that lower socioeconomic position was associated with worse mental health outcomes, though some found no significant effects. One study found that autistic children from below-median income households exhibited significantly higher anxiety symptoms compared to those from above-median income households from the acute to resurgence phase in the United Kingdom (*β* = 2.52, 95% CI [0.22, 4.82], *p* <.05). Analyses accounted for potential confounders including age, sex, ethnicity, school type, and household income [30].

In CYP with elevated emotional and behavioural symptoms, lower household income was associated with worse mental health outcomes during the pandemic. In China, children from lower-income households exhibited significantly greater increases in depressive (RRR = 1.39, 95% CI [1.05, 1.59], *p* <.05) and anxiety symptoms (RRR = 1.57, 95% CI [1.16, 2.14], *p* <.05) from the pre-pandemic to the acute phase, after only adjusting for sex and school level [32]. In the United States, children from low-income households had a higher prevalence of elevated poor mental health symptoms (APR = 1.5, 95% CI [1.0, 2.2], *p* <.05) between the remission and resurgence phases, with analyses adjusting for sex, grade level, and health insurance status [29].

In contrast, two studies from the United Kingdom and Canada observed no significant effects of family income on depressive symptoms in autistic children and those with internalising symptoms across pandemic phases. One study found no significant association between family income and depressive symptoms in autistic children during the acute phase (*β* = 0.32, 95% CI [−0.58, 1.22], *p* >.05); analyses adjusted for sociodemographic factors but not for baseline symptom severity [30]. Similarly, another study reported no significant effects of income on depressive (*β* = − 0.88, SE = 1.54, *p* =.57) and anxiety symptoms (*β* = − 0.12, SE = 0.47, *p* =.81) after adjusting for baseline symptom levels in children with internalising difficulties from the acute to the resurgence phase [28].

Two studies investigated the association between socioeconomic factors and emotional and behavioural difficulties during the remission and resurgence phases of the pandemic. Estrada-Prat and colleagues [33] found no significant associations between SES and emotional and behavioural symptoms in Spain (*β* = –0.30 to 0.87, all *p* >.05); the study did not specify whether baseline symptoms or other confounders were adjusted for. In contrast, Oblath and colleagues [34] reported that increased difficulty paying bills was significantly associated with heightened emotional and behavioural symptoms in the United States during these phases after adjusting for other sociodemographic factors (*p* <.001).

#### Neurodevelopmental symptoms

Two studies examined the effects of socioeconomic position on neurodevelopmental symptoms. The studies included autistic children (one) and those with ADHD (one). One study used clinician-reported data, and another used parent-reported data.

Both studies conducted unadjusted analyses [35, 36].

For ASD, one study reported a significant increase in autism symptom severity among autistic children from low-income families compared to those from higher-income households during the acute and remission phases in Turkey in unadjusted analyses (*t*(40) = −3.09, *p* <.001) [35]. For ADHD, analysis unadjusted for baseline symptoms found no significant associations between SES and ADHD severity (*p* >.05) when comparing pre-lockdown and post-lockdown assessments in children with ADHD in India [36].

#### Other symptoms

One study investigated the effects of socioeconomic position on other symptoms. The study included CYP with post-traumatic stress and obsessive-compulsive symptoms and used parent-reported data. Financial hardship was significantly associated with worse post-traumatic stress (*β* = −2.540, *p* <.05) and obsessive-compulsive symptoms (*β* = −3.248, *p* <.05) among children aged 6–18 with pre-existing mental health and neurodevelopmental conditions during the acute phase compared to pre-pandemic levels in Italy [37]. The analyses adjusted for age, diagnosis, and baseline mental health symptoms, and negative coefficients correspond to greater symptom severity based on the study’s scoring direction.

## Discussion

### Summary of findings

We found mixed evidence for the associations between ethnicity and socioeconomic position with longitudinal mental health outcomes in CYP with pre-existing conditions during the pandemic. We found limited evidence linking ethnicity with internalising symptoms, where most studies reported no significant difference in depressive and anxiety symptoms across all pandemic timepoints. For socioeconomic position, there was evidence that low family income and financial hardship were associated with worse internalising, ASD, ADHD, post-traumatic stress, and obsessive-compulsive symptoms across pandemic phases. However, findings varied due to high heterogeneity in study settings, sample characteristics, and mental health outcomes. No included studies examined ethnicity or socioeconomic position in relation to externalising symptoms. Differences in the socioeconomic indicators (e.g., SES, family income, and financial hardship) may have contributed to inconsistent results across studies, confounding overall subgroup trends.

### Comparisons with other studies

The findings of our review differ from earlier COVID-19 research, which often reported strong associations between ethnicity and mental health outcomes [38, 39]. Guan and colleagues [19] examined CYP from the general population without pre-existing conditions and found mixed results, largely because most studies did not account for social factors alongside ethnicity. They reported that minoritised groups faced similarly elevated risks of internalising symptoms and conduct problems as White children, but key drivers such as deprivation and socioeconomic status were often unmeasured. In contrast, another study identified ethnic differences in emotional and behavioural symptoms in a clinical sample of CYP even after adjusting for deprivation [39]. They found that CYP with ASD and ADHD were most adversely affected during lockdown, showing greater behavioural difficulties than those with emotional disorders, highlighting the need for tailored support for this clinical group and their families. The limited evidence identified in our review may reflect the lack of stratification by ethnic group in clinical samples, consistent with broader evidence of underreporting, where 72% of mental health studies omitted ethnicity data, and only half of those subsequently analysed it [40, 41]. Additionally, barriers to accessing mental health care, such as limited knowledge about services and complicated access pathways, are well-established in CYP from minoritised ethnic groups [41, 42].

Strong evidence linked pandemic-related income loss and poorer mental health outcomes during the pandemic in clinical groups of CYP. Financial hardship and low family income were associated with worse internalising, autistic, and other symptoms. Aligning with previous research, Scrimin and colleagues [44] demonstrated that sudden income drops heightened parental stress and disrupted family routines, while Barroso and colleagues [45] reported disproportionate impacts on neurodivergent children via reduced emotional availability and heightened conflict. Low income may also limit access to healthcare and resources essential for child wellbeing [46], though mechanisms may differ by country depending on healthcare provision. In countries with free healthcare, disparities may still emerge in access and quality, particularly for disadvantaged groups in deprived areas [47, 48]. This variation underscores the need for country-specific analyses when assessing how socioeconomic disparities shape CYP’s mental health.

Measurement heterogeneity may explain the lack of clear associations between SES and mental health in our review, diverging from prior work identifying SES as a key predictor during the pandemic [49] and in clinical samples [50]. One possible explanation is that the wide range of SES measures across studies, such as parental education, occupation, financial stress, and household income or hardship, hindered our ability to consistently measure socioeconomic disadvantage. Elgar and colleagues [51] suggested that different SES indicators tap into separate “active ingredients,” leading to variable associations with mental health. Most included studies used objective or parent-reported measures, which may miss adolescent-specific psychosocial dimensions such as perceived social status and economic insecurity [52]. In contrast, subjective SES reported by CYP may better capture these experiences and explain mental health disparities [53]. Incorporating subjective SES alongside objective measures would thus offer a more complete picture of how socioeconomic factors influence CYP mental health.

Our findings may also reflect broader socioeconomic patterns, where disparities in mental health reflect individual and systemic factors, including housing or job insecurity, exacerbated by the pandemic [54, 55]. A prospective cohort study showed that childhood housing insecurity, such as frequent moves, overcrowding, or unstable living situations, is associated with higher anxiety and depressive symptom scores in both childhood and adulthood, even after adjusting for poverty [56]. Moreover, the stress of eviction or risk of losing housing has been linked to a 4–35% increase in children’s odds of experiencing mental health problems [57]. These patterns indicate that both individual and structural factors shape the impact of socioeconomic inequalities in CYP mental health during the pandemic.

Externalising symptoms were overlooked in the literature on the mental health of CYP with pre-existing conditions during the pandemic. In our review, none of the included longitudinal studies examined how ethnicity or socioeconomic position related to externalising symptoms. This is a significant gap, as externalising difficulties, namely behavioural and conduct problems, are common in children with mental health and neurodevelopmental conditions [58, 59]. Moreover, socioeconomic disadvantage has been consistently linked to elevated levels of aggression, impulsivity, and rule-breaking behaviours in childhood, with lasting impacts on adulthood [60]. Given the absence of data in the included studies, it is unclear whether similar structural disparities were also seen during the pandemic in CYP with pre-existing conditions.

None of the studies in our review investigated how ethnicity and socioeconomic position intersect to influence mental health outcomes in CYP during the pandemic. Yet prior work indicates that these intersections can both exacerbate and buffer risk via social determinants such as poverty, food insecurity, and community marginalisation [61]. Consistent with this, populations from minoritised ethnic backgrounds and economically disadvantaged families faced greater mental health challenges overall [62, 63]. For example, Wright and colleagues [64] found that adolescents from minoritised groups reported heightened anxiety, depression, academic stress, loss of motivation, and loneliness during COVID-19. Together, these findings emphasise the necessity of examining ethnicity and socioeconomic factors jointly to fully understand their combined impact on CYP’s mental health.

Qualitative research may provide insights into the mechanisms underlying the role of ethnicity and socioeconomic position on mental health in CYP. Lee and Wong [65] highlighted the challenges faced by adolescents from socioeconomically deprived and ethnically diverse areas in the United Kingdom, linking barriers in education and social support to poorer mental health. Limited academic opportunities not only affect child and adolescent development but also restrict essential social networks, while environmental stressors like overcrowding and crime further exacerbate distress. Similarly, interviews with autistic children from minoritised ethnic and low-income backgrounds revealed heightened depression and anxiety due to disrupted routines and limited support [66]. These findings underscore how the pandemic not only introduced new challenges but also exacerbated pre-existing vulnerabilities by disrupting access to schools and essential social services.

### Strengths and limitations

We used a rigorous methodological approach by including only longitudinal prospective data to minimise biases from cross-sectional and retrospective studies. We comprehensively reviewed and synthesised heterogeneous findings across longitudinal studies from pre-pandemic to 2021, providing insight into the complex role of ethnic and socioeconomic inequalities on longitudinal mental health outcomes during the pandemic in this clinical population.

There are limitations that should be considered in our included studies. First, there was a paucity of studies that examined the association between ethnicity, socioeconomic position, and mental health outcomes, specifically in clinical groups of CYP. Consequently, we were only able to conduct a narrative synthesis rather than a meta-analysis. For example, no studies investigated the relationship between ethnicity and externalising symptoms. Very few studies incorporated pre-pandemic mental health assessments, and only a small subset adjusted for baseline symptom levels. This limited the extent to which observed differences can be attributed to pandemic-related changes rather than pre-existing inequalities, regression to the mean, or seasonal variation, and constrains the causal interpretation of longitudinal trends. Additionally, the included studies did not examine functional outcomes, including adaptive functioning, academic engagement, daily living skills, and social participation, which represent important domains of development and may complement symptom-based assessments but were beyond the scope of this review. Moreover, because pandemic-related exposures (e.g., infection risk, social restrictions, educational disruption, and financial strain) occurred concurrently and were measured at broad timepoints rather than as discrete events, it was not possible to determine the independent effects of specific pandemic-related factors on CYP’s mental health trajectories.

Second, studies varied widely in how socioeconomic indicators were defined and measured. Indicators ranged from household income to self-reported socioeconomic status and specific financial difficulties, with many relying on parental report. Differences in how families interpret and report financial strain may introduce measurement variability, potentially influencing associations with mental health outcomes. Furthermore, studies differed in the statistical effect sizes reported and in the covariates included in adjusted models, which introduced methodological heterogeneity and limited the comparability of findings across studies.

Third, most samples comprised predominantly White and middle-class participants, resulting in limited representation of minoritised ethnic groups and socioeconomically disadvantaged CYP. This under-representation likely reduced the statistical power to detect inequalities and may have underestimated the true magnitude of socioeconomic and ethnic disparities. Additionally, many studies had relatively small sample sizes, increasing the risk of bias and reducing the reliability of the observed associations.

Finally, more longitudinal studies are needed to capture effects beyond early 2021 and provide an opportunity for meta-analysis of effects. It is crucial to include more data on the mental health outcomes among marginalised groups, as current evidence may have overlooked potentially heterogeneous effects due to unrepresentative samples. Future studies should aim to involve more diverse populations to accurately capture the full scope of disparities in mental health outcomes during and beyond the pandemic. These recommendations align with research priorities informed by children and young people with lived experience [60].

### Recommendation for future research

Future research should prioritise standardised reporting and analysis of ethnicity and socioeconomic position data to better understand how these factors intersect with pre-existing mental health and neurodevelopmental conditions in CYP. A more comprehensive approach that integrates both qualitative and quantitative methodologies is also needed to understand the mechanisms driving these inequalities. Qualitative studies can provide deeper insights into coping strategies and the nuanced ways, in which socioeconomic and ethnic disparities impact mental health outcomes. Additionally, more longitudinal studies are needed to capture effects beyond early 2021 and provide an opportunity for meta-analysis of effects. It is crucial to include more data on the mental health outcomes among diverse and marginalised groups, as current evidence may have overlooked potentially heterogeneous effects due to unrepresentative samples. Furthermore, future studies should incorporate pre-pandemic assessments and consistently adjust for baseline mental health to clarify how socioeconomic and ethnic inequalities evolved during and after the pandemic. There is also a need to examine functional outcomes of CYP to provide a more holistic understanding of how inequalities affect multiple domains of development. These recommendations align with research priorities informed by children and young people with lived experience [67].

## Conclusion

Our systematic review and narrative synthesis found some evidence for the association between socioeconomic position and mental health outcomes during the pandemic in CYP with pre-existing mental health and neurodevelopmental conditions, but limited evidence for ethnicity. The limited exploration of ethnicity underscores the need for improved reporting of social determinants, alongside longitudinal research to examine how ethnicity and socioeconomic position collectively influence the long-term mental health outcomes of CYP with pre-existing conditions. These findings should inform public health policies aimed at reducing racial and social inequalities, strengthening mental health support for vulnerable groups, and guiding targeted preventative interventions for future pandemics, crises, and ongoing recovery efforts.

## Supplementary information

Below is the link to the electronic supplementary material.

Supplementary Material 1

## Data Availability

No datasets were generated or analysed during the current study.

## Declarations

This paper represents independent research is funded by the UK Medical Research Council (MR/W006820/1) and King’s College London member of the MRC Doctoral Training Partnership in Biomedical Sciences and the NIHR Maudsley Biomedical Research Centre at South London and Maudsley NHS Foundation Trust and King’s College London. The views expressed are those of the author(s) and not necessarily those of the NIHR or the Department of Health and Social Care.
