# Supplementary material for: Ethnic and socioeconomic inequalities in the mental health of children and young people with pre-existing mental health and neurodevelopmental conditions during the COVID-19 pandemic: a systematic review of longitudinal studies
Source: Eur Child Adolesc Psychiatry. 2026 Jan 31;35(5):1589–603. doi: 10.1007/s00787-026-02971-2 (PMC13272600; doi:10.1007/s00787-026-02971-2)
Supplement: Supplementary file 1 — Supplementary Material 1 [file 787_2026_2971_MOESM1_ESM.docx]

**Appendix S1**

**PECO elements**

| **Element** | **Description** |
| --- | --- |
| P (Population) | - Children and young people (aged 3-18) with mental health or neurodevelopmental conditions (clinically diagnosed, validated measure, or attending mental health services) |
| E (Exposure) | - The COVID-19 pandemic |
| C (Comparator/Control) | - Not applicable; internal comparators defined by individual studies |
| O (Outcome) | - Changes in mental health symptoms pre- and during pandemic and/or between during pandemic phases |

**Appendix S2**

**Search terms**

Databases searched: OVID Medline, EMBASE, APA PsycInfo, and Global Health between 1^st^ of January 2020 and 25^th^ of November 2025

| **#** | **Search terms** |
| --- | --- |
| 1 | (child* or adolesc* or teen* or "young people" or "young person" or youth) |
| 2 | ('mental disorder" or "mental illness" or "emotional difficulties" or "emotional disorder" or behavio* or conduct or neurodevelopmental or autis* or "attention deficit" or adhd） |
| 3 | (observational or longitudinal or cross-sectional or cohort or case-control) |
| 4 | (COVID or SARS-CoV or coronavirus) |
| 5 | #1 AND #2 AND #3 AND #4 |

**Appendix S3**

**Risk of bias indicators**

Three study quality appraisal and risk of bias assessment scales (i.e., Newcastle-Ottawa Scale for Non-randomised Studies, National Institute of Health Quality Assessment Tool for Observational Cohort and Cross-sectional Studies, and Critical Appraisal Skills Programme Checklist for Cohort Studies) were reviewed to develop a list of risk of bias indicators to suit our research questions and included studies:

1. Sampling and recruitment - Were sampling methods used to achieve a representative sample of the population being studied, e.g., use of quota sampling or probability sampling to reflect demographics of wider population?
2. Sample size - Was a sample size justification, power description, or variance and effect estimates provided?
3. Survey delivery - Was survey delivery mode consistent across data collection timepoints, e.g., in-person or online?
4. Outcome assessment - Were measures of mental health outcomes collected more than 12-months apart from each other?
5. Attrition - Was attrition/loss to follow-up after baseline 20% or less and were appropriate strategies used to address attrition, e.g., analysis of whether attrition occurred at random and if not, strategies for selective attrition, such as weighting?
6. Confounding - Were confounding factors considered in the design and/or analysis, e.g., adjusted statistically?

Overall risk of bias: A rating of high, moderate/unclear, low, and not applicable were given for each indicator, where lower ratings indicated lower risk of bias. An overall quality rating was given for each study, where a study would be rated an overall high risk of bias if at least two indicators were rated high without any rated low, moderate if there were any indicators rated medium or high without meeting an overall high risk of bias, and low if all indicators were rated low.

**Appendix S4**

**Traffic light plot of risk of bias
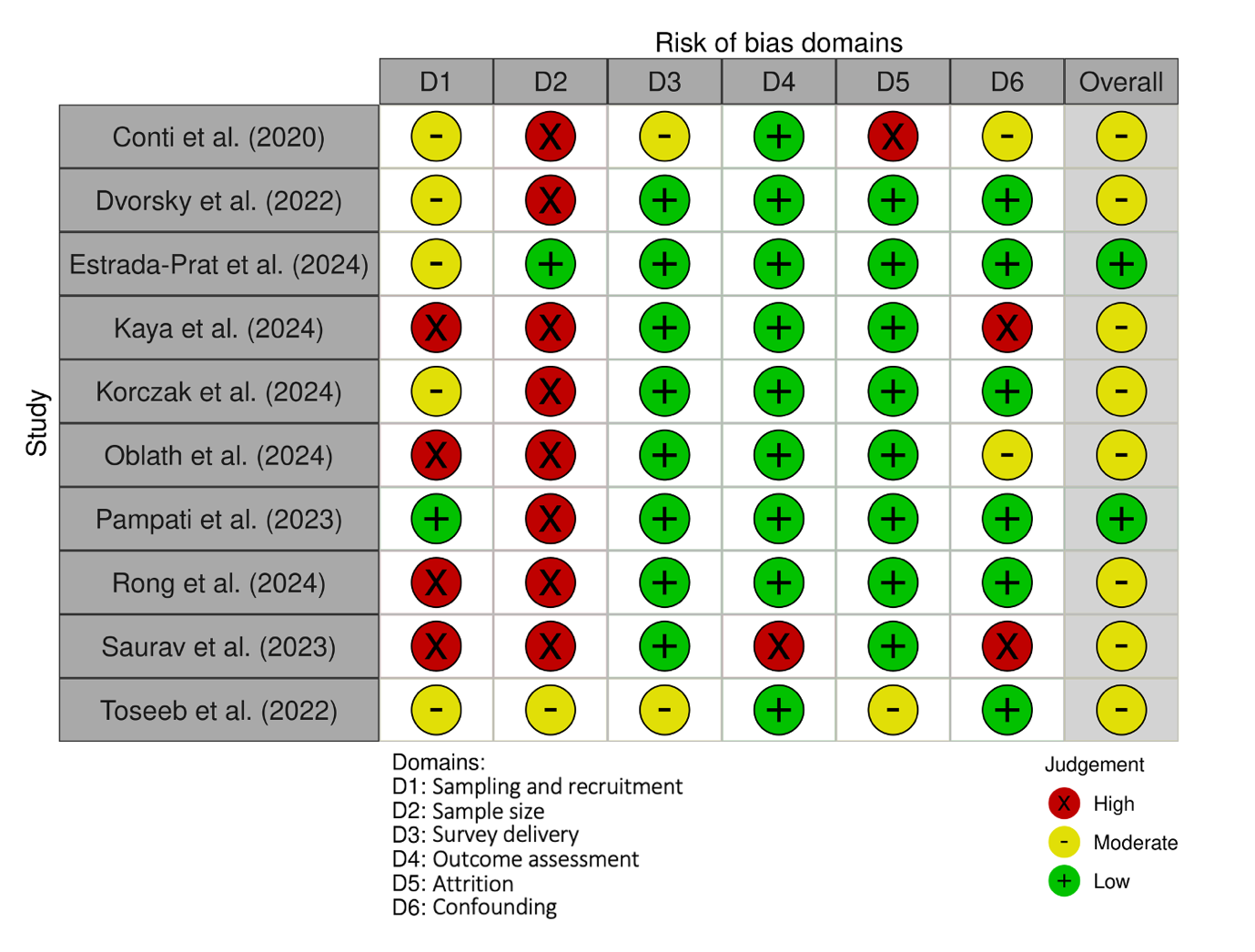
assessments**
